# Mitochondrial SLC3A1 regulates sexual dimorphism in cystinuria

**DOI:** 10.1016/j.gendis.2024.101472

**Published:** 2024-11-29

**Authors:** Jingyi Su, Yongdong Pan, Fengbo Zhong, Yi Zhong, Jiaxin Huang, Shengnan Liu, Kaiyuan Wang, Kai Lin, Xiangchen Gu, Dali Li, Qihui Wu, Hongquan Geng, Yuting Guan, Guofeng Xu

**Affiliations:** a Department of Pediatric Urology, Xinhua Hospital Affiliated to Shanghai Jiao Tong University School of Medicine, Shanghai 200092, China; b Shanghai Frontiers Science Center of Genome Editing and Cell Therapy, Shanghai Key Laboratory of Regulatory Biology, Institute of Biomedical Sciences and School of Life Sciences, East China Normal University, Shanghai 200241, China; c Department of Nephrology, Yueyang Hospital of Integrated Traditional Chinese and Western Medicine, Shanghai University of Traditional Chinese Medicine, Shanghai 200437, China; d Shanghai Key Laboratory of Anesthesiology and Brain Functional Modulation, Clinical Research Center for Anesthesiology and Perioperative Medicine, Translational Research Institute of Brain and Brain-Like Intelligence, Shanghai Fourth People's Hospital, School of Medicine, Tongji University, Shanghai 200434, China; e Department of Urology, Children's Hospital of Fudan University, Shanghai 201102, China; f Chongqing Key Laboratory of Precision Optics, Chongqing Institute of East China Normal University, Chongqing 401120, China

**Keywords:** Cystinuria, Kidney injury, Mitochondria, Sex bias, Slc3a1

## Abstract

Cystinuria is the most common inheritable cause of kidney stone disease, with males exhibiting a higher susceptibility than females. However, the cellular origin and underlying mechanisms of sex differences in cystinuria remain elusive. This study aims to investigate the mechanism using *Slc3a1* knockout mice. We found that male mice lacking the *Slc3a1* gene exhibited more severe stone formation and renal injuries, unaffected by double knockout of another sex-dependent-expressed cystine transporter *Slc7a13* or orchidectomy procedure. Further investigations revealed aberrant mitochondrial functions as the primary factor contributing to the severity of cystinuria in *Slc3a1* knockout male mice. Mechanistically, higher SLC3A1 levels in male kidneys could enhance mitochondrial functions through modulation of mitochondrial NAD^+^ uptake primarily in proximal tubule cells. Supplementation with an NAD^+^ precursor rescued the sex differences caused by *Slc3a1* knockout. Our studies uncover the crucial role of *Slc3a1* in mitochondrial functions and provide novel insights into potential interventions for sexual dimorphism of cystinuria.

## Introduction

Cystinuria is an inherited disorder characterized by impaired reabsorption of cystine and dibasic amino acids in the renal proximal tubules.^1^ It affects approximately 1 in every 7000 neonates, making it the most common genetic cause of renal stones in children and accounting for 10% of all cases of pediatric nephrolithiasis.^2^ Up to 70% of patients with cystinuria may develop chronic kidney disease, which can progress to end-stage renal disease.^3^ Despite efforts towards finding a cure for cystinuria, only a few drugs have been approved so far.

Increasing evidence suggests that renal function decline associated with aging is more rapid in men versus women, as observed in human kidney studies. Renal injuries tend to be more aggressive and progress to end-stage renal disease at a faster rate in men than in women.^4^^,^^5^ Similarly, cystinuria exhibits a sex-dependent response, with males experiencing an earlier onset of stone formation and a higher number and larger size of stones.^6^ Despite these sex differences, the mechanisms underlying sexual heterogeneity in cystinuria remain unclear. Gaining a better understanding of the molecular controls involved will advance our comprehension of ongoing clinical trials related to drug usage, dosage, and effectiveness between sexes since women experience adverse drug reactions nearly twice as often as men.^7^

Cystine is a homomeric amino acid containing sulfur and consists of two cysteine molecules linked by a disulfide bond. It is freely filtered and reabsorbed in the renal system through a transport mechanism involving the heterodimeric complex b(0,+)AT (encoded by SCL7A9) and rBAT (encoded by SLC3A1).^8^ Mutations in these genes impair l-cystine transportation in renal proximal tubules.^9^ AGT1 (encoded by SLC7A13) has been identified as a new partner of rBAT in the S3 segment. While transporter b(0,+)AT is located in the S1 segment of proximal renal tubules, rBAT is most abundant in the S3 segment.^10^ In mouse models, S*lc3a1* knockout (KO) exhibits higher penetrance of urolithiasis among males,^11^, ^12^, ^13^ whereas no sex differences were observed regarding stone formation among *Slc7a9* KO mice. Few studies have been published on sex differences related to stone formation involving the novel identified cystine transporter AGT1. The reasons behind these sex differences remain unclear and require further exploration.

There is a high abundance of mitochondria in renal tubular epithelial cells, second only to cardiomyocytes. Mitochondria serve as the “energy factory” for renal tubular epithelial cells, continuously producing ATP to support renal tubule reabsorption function.^14^ Accumulating evidence suggests that mitochondrial dysfunction plays a critical role in the pathogenesis of kidney diseases.^15^ For instance, mitochondrial dysfunction was observed in mouse kidneys within 3 h after glycerol injection, preceding any signs of kidney injury, and persisted for up to 144 h.^16^ Furthermore, persistent impairment of mitochondrial bioenergetics and β-oxidation in renal tissues facilitated the transition from experimental acute kidney injury to chronic kidney disease following folic acid treatment.^17^ Notably, mitochondria exhibit sexual dimorphism primarily involving oxidative capacities, calcium handling, and resistance to oxidative stress.^18^ Increasing evidence suggests that biological sex influences factors crucial for kidney health and contributes to differential injury responses in patients with kidney disease.^19^ There is also growing recognition of significant sex-related differences in mitochondrial morphology, function, and homeostasis, as well as response variations to acute kidney injury^20^ and progression of chronic kidney disease.^21^ The etiology of cystinuria, whether it is attributed to sexual dimorphism of mitochondria, remains unknown.

In this study, our objective was to investigate the underlying mechanism of sex disparities in cystinuria. To address this question, we examined stone formation and kidney injury in *Slc3a1* KO mice, *Slc3a1* and *Slc7a13* double KO mice, as well as orchiectomized *Slc3a1* KO mice. Significant differences between males and females were observed in stone formation and kidney injury in *Slc3a1* KO mice, which were not improved by double KO of *Slc3a1* and *Slc7a13* or orchidectomy. The RNA sequencing and molecular experiments revealed that *Slc3a1* KO male kidneys exhibited abnormal mitochondrial functions. By integrating unbiased bulk RNA sequencing, single-cell RNA sequencing, and molecular experiments, it was found that *Slc3a1* enhanced mitochondrial functions by increasing mitochondrial NAD ^+^ uptake in the proximal tubule. Our findings suggest that mitochondria in the proximal tubule are a key factor in understanding sex disparities in cystinuria pathologies.

## Materials and methods

### Mice

C57BL/6 mice were used in this study. All mice were maintained under specific pathogen-free conditions with ambient temperature 20–22 °C, humidity 50–70%, and a 12 h/12 h light/dark cycle. *Slc3a1* mutant mice and *Slc7a13* mutant mice were generated by co-injection of Cas9 mRNA and sgRNA in Cyagen Biosciences. The sgRNA sequences and genotyping primers are listed in Table S1.

Kidney, serum, and urine were collected from 8-week-old and 20-week-old wild-type (WT) mice and *Slc3a1* KO mice (or *Slc7a13* and *Slc3a1* double KO mice) for analysis. For orchiectomy, *Slc3a1* cystinuria males were conducted according to previous protocols.^22^ In brief, mice were anesthetized with tribromoethanol prior to performing a single incision on the scrotum. Subsequently, the cremaster muscles were dissected, exposing the testicular region where ligatures were applied around the blood vessels and vas deferens. The blood vessels, cauda, and caput epididymis were carefully severed before removing the testis. Finally, the remaining contents were replaced and the skin was closed using metal clips. Mice receiving nicotinamide mononucleotide (NMN) treatment had a daily intraperitoneal injection (500 mg/kg).

### NAD^+^ measurement in extracts

For cells, the culture medium was removed and ice-cold NAD^+^/NADH extraction buffer was added. The cells were gently pipetted to promote cell lysis on ice or at room temperature and then centrifuged at 12,000 *g* and 4 °C for 10 min. The supernatant was collected for subsequent assay. NAD^+^ measurement was performed using the NAD^+^/NADH Assay Kit with WST-8 (Beyotime #S0175) according to the manufacturer's instructions.

### Mitochondrial isolation

Cells were washed with phosphate buffer saline, digested with trypsin-EDTA solution, and centrifuged at 100–200 *g* at room temperature for 5–10 min. The cells were gently resuspended in ice-cold phosphate buffer saline; a small number of cells were taken for counting, and the remaining cells were centrifuged at 600 *g* at 4 °C for 5 min. After the supernatant was discarded and 2 mL of mitochondria isolation reagent from the Cell Mitochondria Isolation Kit (Beyotime #C3601) per 2 × 10^7^ cells were added. The cells were then gently resuspended and placed on ice for 10–15 min. The cell suspension was transferred to a glass homogenizer and homogenized 45 times. The cell homogenate a centrifuged at 600 *g* and 4 °C for 10 min. The supernatant was carefully transferred to a new centrifuge tube and then centrifuged at 11,000 *g* and 4 °C for 10 min. The supernatant was carefully removed to obtain mitochondria in the pellet.

### Mammalian mitochondrial NAD^+^ uptake

To measure mitochondrial NAD^+^ uptake, isolated mitochondria were resuspended in MiR05 containing 10 mM pyruvate along with NAD^+^ (1 mM) in a 1.5 mL Eppendorf tube. The reaction was rotated and the tube was briefly opened every 10 min to allow for re-oxygenation for 1 h. Mitochondria were pelleted by centrifugation (14,000 *g* for 2 min). The mitochondrial pellet was washed 2 times with ice-cold mitochondrial isolation buffer and then lysed for biochemical measurements of mitochondria NAD^+^ content.

### Immunofluorescence staining

Cells were washed with phosphate buffer saline, fixed with 4% paraformaldehyde, permeabilized with phosphate buffer saline-0.2% Triton X 100, and blocked with 5% fetal bovine serum. Immunostaining was performed using the primary antibodies against SLC3A1 (Proteintech #19912-1-AP) and ATP5A1 (Proteintech #66037-1-Ig).

### Real-time reverse-transcription PCR

RNA was isolated using TRIzol reagent (Invitrogen), and RNA was reverse-transcribed using the HiScript® III RT SuperMix for quantitative PCR (Vazyme, Nanjing, China). Real-time reverse-transcription PCR was performed with a SYBR Green Master Mix (Applied Biosystems). The primers used are listed in Table S2.

### Western blotting

Kidney tissue or cultured cell lysates were prepared with ice-cold lysis buffer (CST # 9806) containing protease inhibitor cocktail (cOmplete Mini, Roche #11836153001) and a phosphatase inhibitor (PhosSTOP, Roche #4906837001), resolved on 8%–12% gradient gels, transferred on to polyvinylidene difluoride membranes, and probed with the antibodies. The antibodies used are listed in Table S3.

### Urine collection and determination

Urine samples were collected from food-free metabolic cages for 24 h and stored at −80 °C. The concentration of urinary cystine was measured by Pu Luo (Wuhan) Medical Biotechnology Co., Ltd., China The urinary creatinine was measured by Chemray 240 (Wuhan Servicebio Biotechnology Co., Ltd., China) according to the manufacturer's instructions. Finally, the renal cystine concentrations were normalized to creatinine levels. The urinary crystals were examined by concentrating urine through centrifugation at 5000 *g* for 15 min, followed by removal of the supernatant and resuspension with 1/20th of the original volume. Subsequently, direct microscopic observation was conducted.

### Kidney histological analysis

Kidneys were harvested from mice, rinsed in phosphate buffer saline, fixed in 10 % formalin, and embedded in paraffin. Hematoxylin and eosin staining and Sirius red staining were performed for histological analysis. The tubular injury was scored as previously described.^23^ In brief, semi-quantitation was evaluated, including tubular degeneration, interstitial fibrosis, hyaline cast, mononuclear infiltration, and glomerulopathy using the following scoring system: score 0 for no tubular injury; score 1 for injury involving <10% tubules; score 2 for injury involving 10%–25% tubules; score 3 for injury involving 26%–50% tubules; score 4 for injury involving 51%–74% tubules; and score 5 for injury involving >75% tubules.

### RNA sequencing

For bulk RNA sequencing, total RNA was extracted using Trizol reagent (Thermofisher, #15596018) following the manufacturer's procedure. The quantity and purity of total RNA were analyzed by Bioanalyzer 2100 and RNA 6000 Nano LabChip Kit (Agilent, CA, USA, 5067-1511). High-quality RNA samples with an RIN number >7.0 were used to construct a sequencing library. The RNA libraries were sequenced on the Illumina Novaseq™ 6000 platform by LC Bio Technology CO., Ltd. (Hangzhou, China). For single-cell RNA sequencing, single-cell suspensions were loaded to 10× chromium according to the manufacturer's instructions for the 10× Genomics Chromium Single-Cell 3′ Kit (V3). The following cDNA amplification and library construction steps were performed according to the standard protocol. Libraries were sequenced on an Illumina sequencing system (paired-end multiplexing run, 150 bp) by LC-Bio Technology Co., Ltd. (Hangzhou, China) at a minimum depth of 20,000 reads per cell.

### Mitochondrial DNA measurement

The genomic DNA of single-cell suspension of kidneys was extracted using the TIANamp Genomic DNA Kit (TIANGEN China). Quantitative PCR was performed with a SYBR Green Master Mix (Applied Biosystems). The mtDNA/nDNA ratio was normalized and calculated using the ΔΔCt method. Finally, the data were analyzed using GraphPad Prism. The primers used are listed in Table S2.

### Transmission electron microscopy

Transmission electron microscopy was used to examine mitochondrial structure. First, fresh kidneys were fixed in 4% glutaraldehyde for 12 h, post-fixed in 1% osmium tetroxide for 1 h, dehydrated by ethanol, penetrated with acetone, and finally embedded in resin. Ultrathin slices (40–50 nm) were stained with lead citrate and uranyl acetate before analysis by an electron microscope (Hitachi, Tokyo, Japan).

### ATP and GSH assay

Kidney tissues from 8-week-old male (*n* = 4) and female (*n* = 4) WT mice were used for determining the content of ATP and GSH with the ATP Assay Kit (Beyotime, China) and the GSH and GSSG Assay Kit (Beyotime, China) according to the manufacturer's instructions.

### Cell culture

For primary culture of renal tubule cells, kidneys were collected from 2- to 4-week-old male mice. Cells were isolated by 2 mg/mL collagenase I (Gibco #17018-029) digestion at 37 °C with gentle stirring for 30 min and filtered through a 100-mm mesh. The cell suspension was cultured in RPMI 1640 (Corning #10-040-CM) supplement with 10% fetal bovine serum (Atlanta Biologicals #S11950), 20 ng/mL EGF (Peprotech #AF-100-15), 1 × ITS (Gibco #51500–056), and 1% penicillin-streptomycin (Corning #30-002-CI) at 5% CO_2_ and 37 °C.

### Statistical analysis

An unpaired student's *t*-test was used to compare differences between two groups and one-way ANOVA was used to compare intergroup differences between three or more groups. Results were presented as mean ± standard error of the mean and *p* values < 0.05 were considered statistically significant. Statistical data and figures were performed with GraphPad Prism 9.

## Results

### Male *Slc3a1* KO mice exhibited severer stone formation and renal injury

We first generated *Slc3a1* KO mice by deleting exon 2–4 using the CRISPR-Cas9/sgRNA system (Fig. S1A). A line with a confirmed deletion of 6830 bp through Sanger sequencing and quantitative PCR (Figs. S1B–D), was selected for subsequent experiments. These findings confirmed the successful generation of *Slc3a1* KO mice.

**Figure 1:**
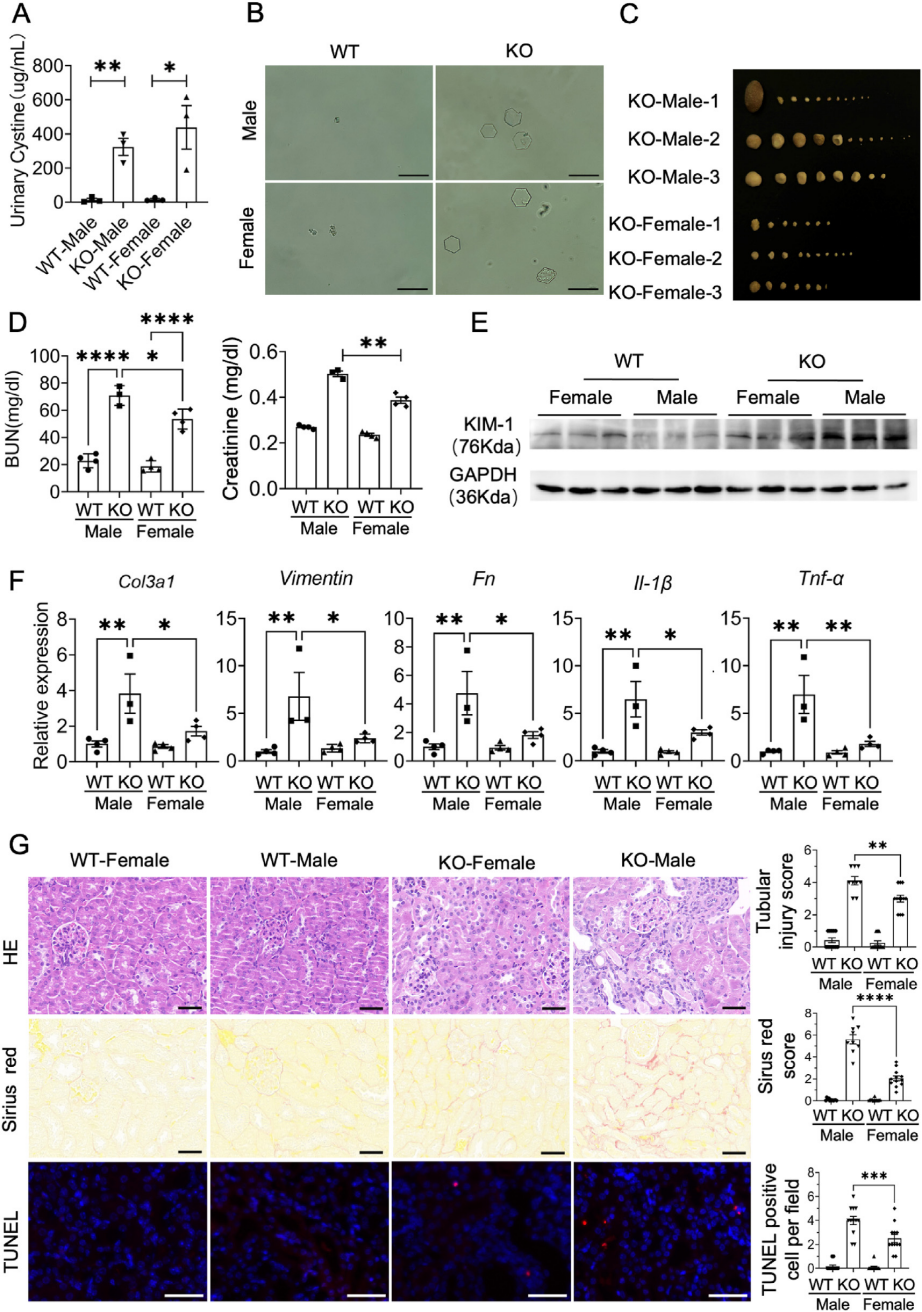
Male *Slc3a1* KO mice exhibited severer stone formation and renal injuries. **(A)** Relative urinary cystine concentration of *Slc3a1* KO and WT mice from both sexes. **(B)** Typical hexagonal cystine crystals from the urine of *Slc3a1* KO and WT mice. Scale bar,100 μm. **(C)** Bladder stones in 20-week-old *Slc3a1* KO mice from both sexes. **(D)** Serum blood urea nitrogen (BUN) and creatinine measurement of *Slc3a1* KO and WT mice from both sexes. **(E)** Protein levels of KIM1 in the kidneys of *Slc3a1* KO and WT mice from both sexes. **(F)** Relative mRNA levels of fibrosis and inflammatory genes (*Col3a1*, *Vimentin*, *Fibronectin*, *IL-1β*, and *TNF-α*) in the kidneys of *Slc3a1* KO and WT mice from both sexes. **(G)** Representative images (left) of hematoxylin/eosin-, Sirius red-, and TUNEL-stained kidney sections of *Slc3a1* KO and WT mice from both sexes and their quantification (right). Scale bar, 100 μm. All data are represented as mean ± standard error mean. *p* values were calculated by one-way ANOVA with the post hoc Tukey test. ∗∗∗∗*p* < 0.0001, ∗∗∗*p* < 0.001, ∗∗*p* < 0.01, ∗*p* < 0.05.

To investigate the susceptibility of males and females to cystinuria, cystine concentration was tested in the urine from both sexes of control and *Slc3a1* KO mice at 20 weeks old. Both male and female mice at 20 weeks old exhibited a significant increase in urinary cystine levels, as well as the presence of cystine hexagonal crystals compared with control littermates (Fig. 1A, B). However, no significant differences were observed between the sexes in urinary cystine or hexagonal crystal levels (Fig. 1A, B). Despite this lack of difference, bladder stones were found in 40% (8 out of 20) *Slc3a1* KO female mice and 65% (13 out of 20) *Slc3a1* KO male mice and female mice exhibited smaller and less severe bladder stones (Fig. 1C), indicating that incidence rate of stone formation was lower in females versus males. In addition, female mice showed a lower increase in serum blood urea nitrogen and creatinine, suggesting better renal function compared with males (Fig. 1D). To further investigate renal damage caused by cystinuria, kidney injury molecule 1 (KIM1), a biomarker of renal injury, was measured. The results indicated a significant increase in KIM1 protein levels in *Slc3a1* KO kidneys compared with WT littermates (Fig. 1E). As expected, fibrotic markers such as *Col3a1*, *Fibronectin*, and *Vimentin*, along with immune markers *IL-1β* and *TNF-α*, showed higher expression levels in *Slc3a1* KO male kidneys versus female kidneys (Fig. 1F). To observe the pathological changes in cystinuria kidneys, hematoxylin and eosin staining, Sirius red staining, and TUNEL staining were performed. Hematoxylin and eosin staining showed more severe epithelial atrophy, tubular dilation, and inflammatory cell infiltration in cystinuria males than females. Sirius red staining and TUNEL analysis suggested that *Slc3a1* KO male mice had a greater degree of fibrosis and cell apoptosis than females (Fig. 1G). Taken together, these findings indicate that *Slc3a1* KO male mice display a more pronounced cystinuria phenotype and renal injury than their female counterparts.

### The male susceptibility to cystinuria is independent of *Slc7a13*

**Figure 2:**
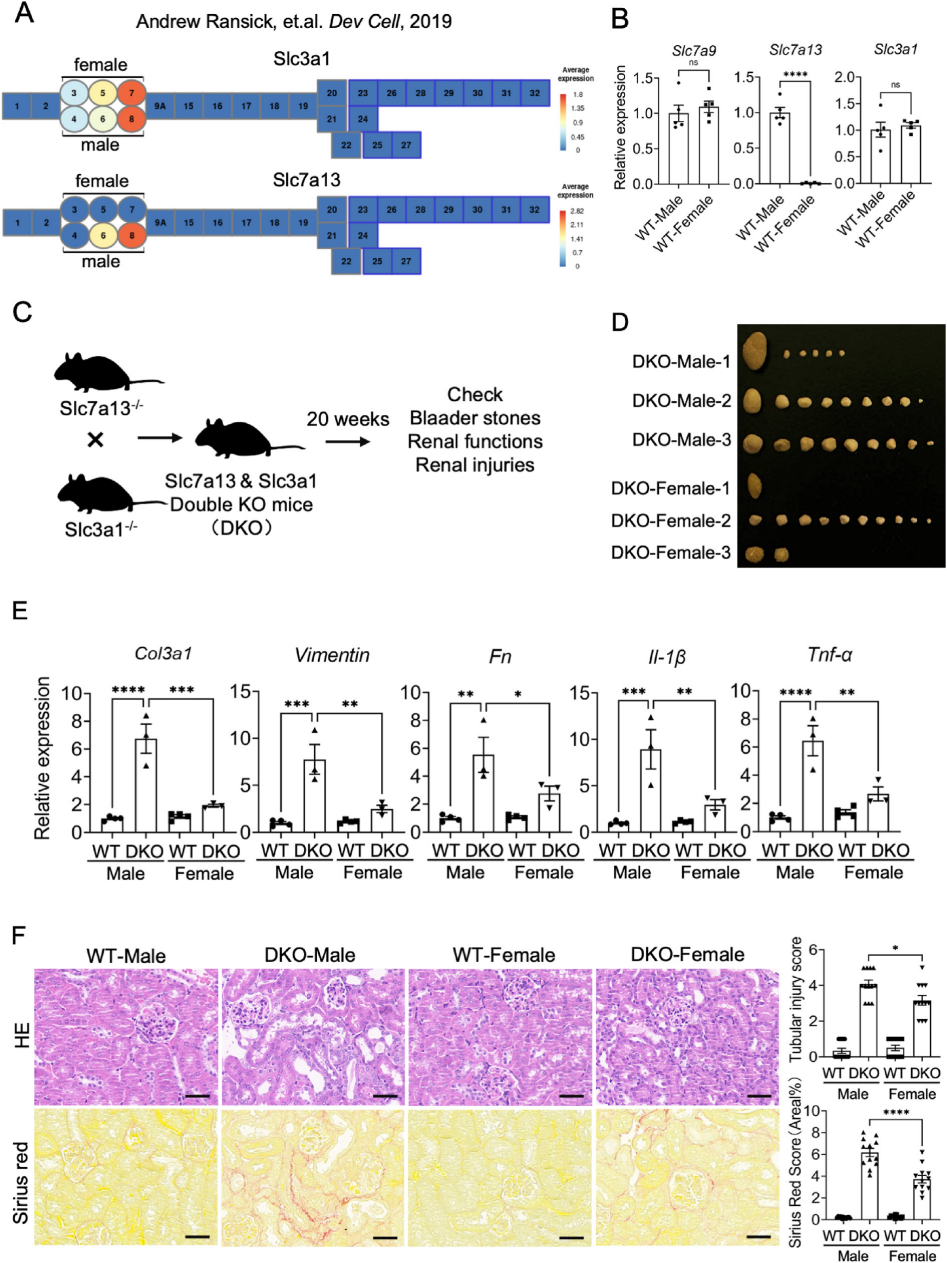
The male susceptibility to cystinuria is independent of *Slc7a13*. **(A)** The published single-cell RNA sequencing data representing the expression levels of *Slc3a1* and *Slc7a13* in mouse kidneys. The highlighted numbers are as below: 3-segment 1 of the proximal tubule (female), 4-segment 1 of the proximal tubule (male), 5-segment 2 of the proximal tubule (female), 6-segment 2 of the proximal tubule (male), 7-segment 3 of the proximal tubule (female), 8-segment 3 of the proximal tubule (male). The annotation of other numbers shown here can be found at https://cello.shinyapps.io/kidneycellexplorer/. **(B)** Relative mRNA levels of *Slc7a9*, *Slc7a13*, and *Slc3a1* in the WT mouse kidneys. **(C)** Schematic diagram of the *Slc7a13* and *Slc3a1* double knockout (DKO) models. **(D)** Bladder stones in 20-week-old DKO mice from both sexes. **(E)** Relative mRNA levels of fibrosis and inflammatory genes (*Col3a1*, *Vimentin*, *Fibronectin*, *IL-1β*, and *TNF-α*) in the kidneys of DKO and WT mice from both sexes. **(F)** Representative images (left) of hematoxylin/eosin- and Sirius red-stained kidney sections of DKO and WT mice from both sexes and their quantification (right). Scale bar, 100 μm. All data are represented as mean ± standard error of the mean. *p* values were calculated by *t*-test or one-way ANOVA with the post hoc Tukey test. ∗∗∗∗*p* < 0.0001, ∗∗∗*p* < 0.001, ∗∗*p* < 0.01, ∗*p* < 0.05.

As we mentioned before, *Slc7a13* was a novel partner of *Slc3a1* in the S3 segment in the proximal tubule,^10^ and most importantly, through the public single-cell RNA sequencing,^24^ the transcript levels of *Slc3a1* were not sex-dependent whereas *Slc7a13* was only expressed in male proximal tubule cells (Fig. 2A). The quantitative PCR results were also confirmed that among the three known cystine transporters, only the expression level of *Slc7a3* was sex-dependent, and it was nearly not expressed in female kidneys (Fig. 2B). We wondered whether the sex-dependent expression of *Slc7a13* was the cause of the sex differences of *Slc3a1* KO induced cystinuria, thus we generated *Slc7a13* KO mice with CRISPR/Cas9 technology (Fig. S2) and crossed them with *Slc3a1* KO mice to make a double KO (DKO) mouse (Fig. 2C). At the age of 20 weeks old, bladder stones were still found from both male and female DKO mice. Similar to the *Slc3a1* KO models, female DKO mice still showed less serious in the sizes and numbers of bladder stones (Fig. 2D). Fibrotic markers *Col3a1*, *Fibronectin*, and *Vimentin*, along with immune markers *IL-1β* and *TNF-α* showed consistently higher expression levels in DKO male kidneys compared with DKO female kidneys (Fig. 2E). Hematoxylin and eosin staining and Sirius red staining also showed more tubule dilation and collagen deposition in DKO male kidneys (Fig. 2F). Altogether, these data showed that even if the expression of *Slc7a13* was higher in male kidneys, knocking out *Slc7a13* cannot rescue the male severity of cystinuria, indicating that *Slc7a13* did not have an impact on the sex difference of cystinuria.

### The orchidectomy procedure failed to mitigate the sexual disparity in cystinuria

**Figure 3:**
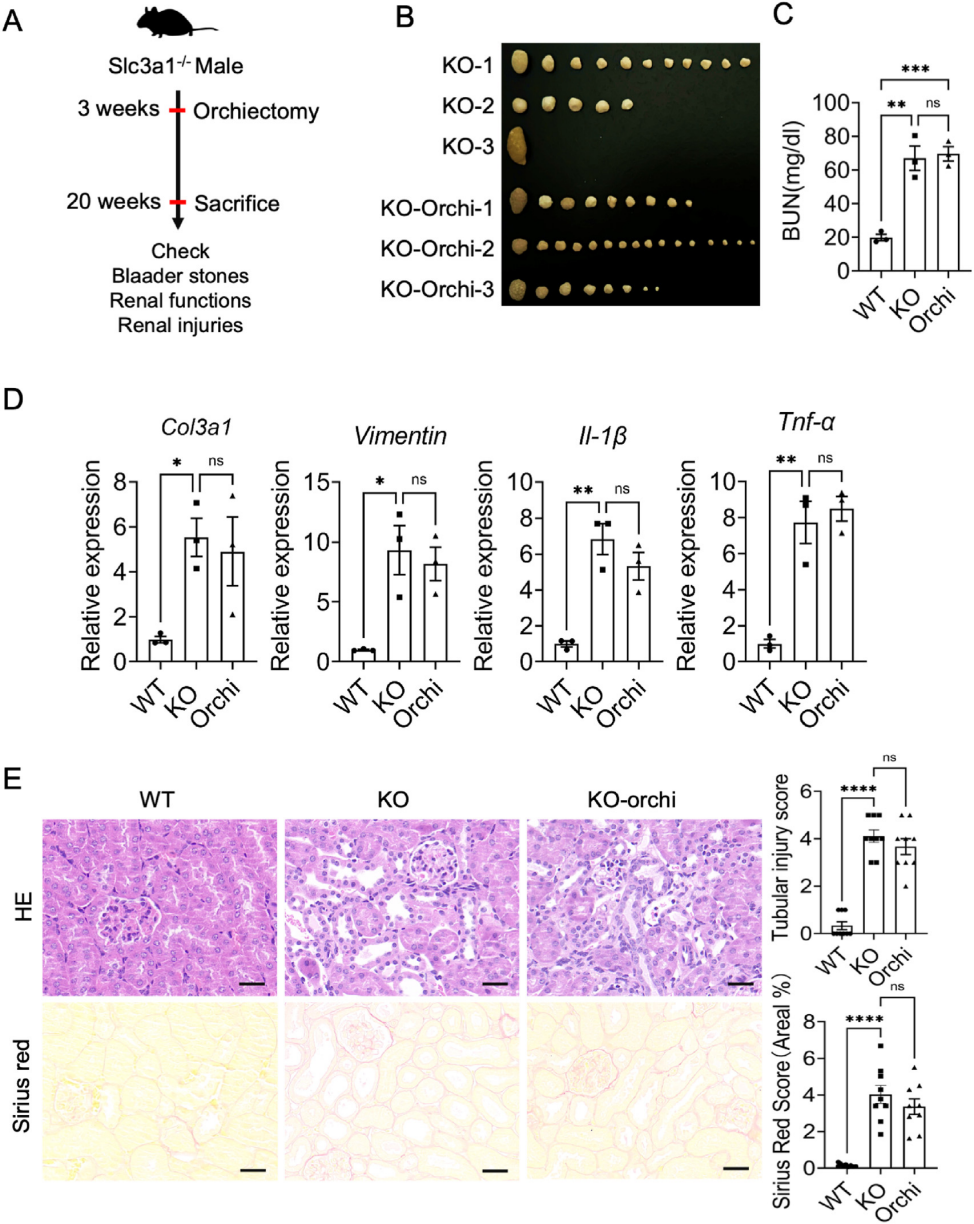
The orchidectomy procedure failed to mitigate the sexual disparity observed in cystinuria. **(A)** Study design of the orchiectomy procedure with *Slc3a1* KO males. **(B)** Bladder stones from control *Slc3a1* KO males and orchiectomized *Slc3a1* KO males at 20 weeks old. **(C)** Blood urea nitrogen (BUN) measurement of WT males, sham *Slc3a1* KO males, and orchiectomized *Slc3a1* KO males. **(D)** Relative mRNA levels of fibrosis and immune markers (*Col3a1*, *Vimentin*, *Fibronectin*, *IL-1β*, and *TNFα*) in the kidneys of WT males, sham *Slc3a1* KO males, and orchiectomized *Slc3a1* KO males. **(E)** Representative images (left) of hematoxylin/eosin- and Sirius red-stained kidney sections of WT males, sham *Slc3a1* KO males, and orchiectomized *Slc3a1* KO males, and their quantification (right). Scale bar, 100 μm. All data are represented as mean ± standard error mean. *p* values were calculated by one-way ANOVA with the post hoc Tukey test. ∗∗∗∗*p* < 0.0001, ∗∗*p* < 0.01, ∗*p* < 0.05.

The sex hormones play a crucial role in the sexual dimorphism of renal structure and function.^25^^,^^26^ Androgens and testosterone have been demonstrated to be involved in regulating the sex differences in kidneys.^27^, ^28^, ^29^ In particular, deleting kidney-specific androgen receptor eliminated the sex differences in renal physiology.^27^ To investigate whether androgens contribute to the sexual dimorphisms of cystinuria, we performed orchiectomy on *Slc3a1* KO male mice at 3 weeks old and sacrificed them at 20 weeks old (Fig. 3A). Surprisingly, there were no significant differences in terms of the numbers and sizes of cystine stones between control *Slc3a1* KO males and orchidectomized *Slc3a1* KO males (Fig. 3B), nor was there any noticeable disparity in blood urea nitrogen concentration between these two groups (Fig. 3C). Moreover, the expression levels of *Col3a1*, *Vimentin*, *TNF-α*, and *IL-1β* were comparable between orchidectomized *Slc3a1* KO males and untreated *Slc3a1* KO males (Fig. 3D). Hematoxylin and eosin staining and Sirius red staining also revealed similar tubule dilation patterns and collagen deposition levels between these two groups (Fig. 3E). In conclusion, these findings indicate that androgens do not influence stone development or renal injury in cystinuria, thus failing to alleviate the sexual difference associated with cystinuria.

### Mitochondria might serve as a central target for the observed sex differences in *Slc3a1* KO mice

**Figure 4:**
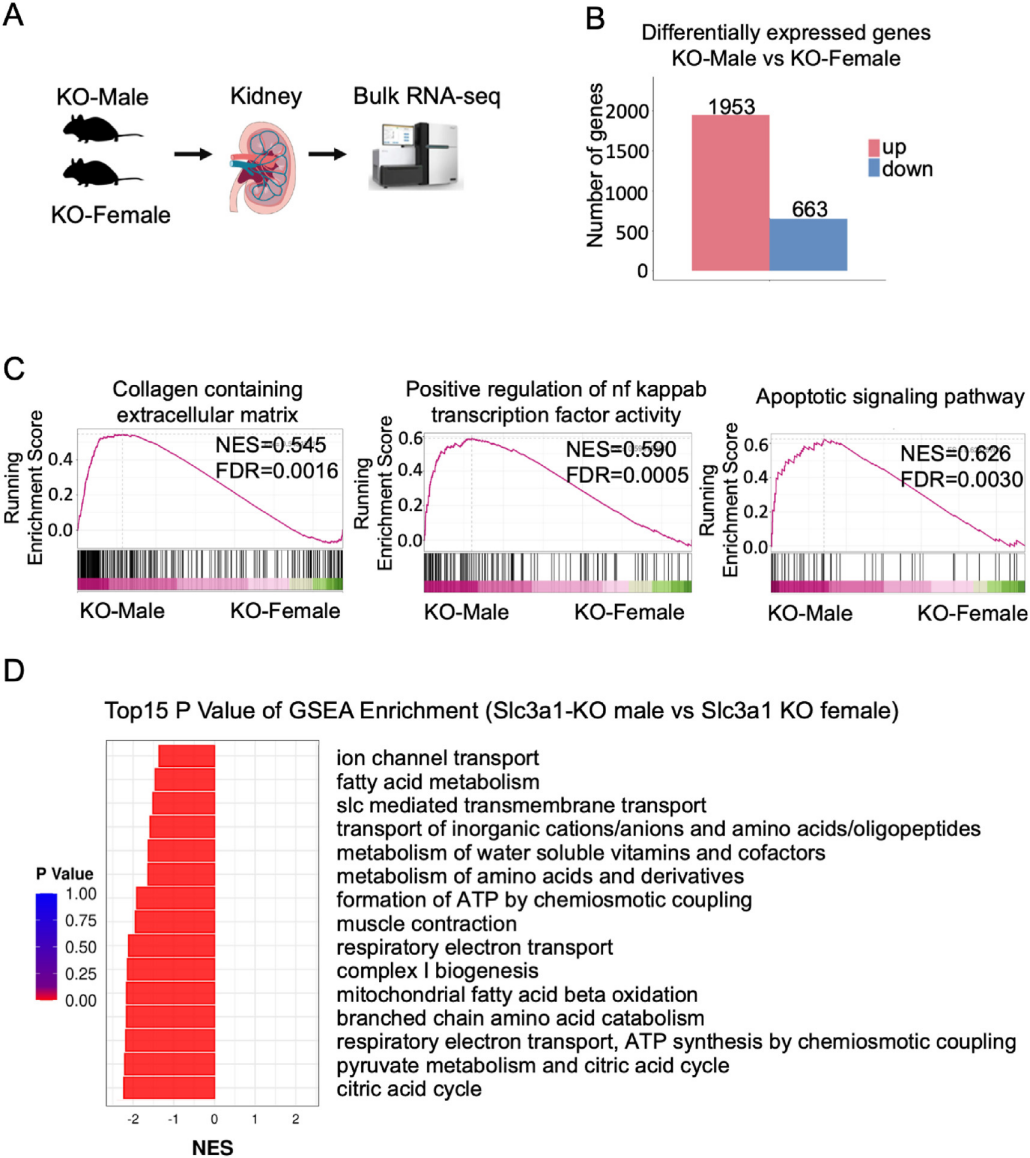
Mitochondria might serve as a central target for the observed sex differences in *Slc3a1* KO mice. **(A)** Schematic diagram of the bulk RNA sequencing (RNA-seq) with kidneys from *Slc3a1* KO females and males. **(B)** Graph of the differentially expressed genes in bulk RNA-seq with 1953 up-regulated (red) and 663 down-regulated genes (blue). **(C)** Gene set enrichment analysis (GSEA) analysis of collagen-containing extracellular matrix, NF-kB signaling, and apoptotic signaling pathways of KO male kidneys over female kidneys from bulk RNA-seq data. NES, normalized enrichment score; Pval, nominal *p*-value. **(D)** The top 15 GSEA enrichment terms with the lowest *p*-values comparing KO male kidneys with female kidneys from bulk RNA-seq data.

Considering that the tested *Slc7a13* and orchidectomy were not the underlying causes of sex differences in cystinuria, we conducted a bulk RNA sequencing on six *Slc3a1* KO kidney samples (3 males and 3 females) to further investigate the underlying mechanism (Fig. 4A). Analysis of KO-male versus KO-female RNA sequencing data revealed a total of 2616 differentially expressed genes, with 1953 up-regulated genes and 663 down-regulated genes (Fig. 4B). GO enrichment analysis confirmed *Slc3a1* KO males exhibited a more pronounced phenotype than females, as evidenced by the enrichment of collagen-containing extracellular matrix, NF-κB signaling, and apoptotic signaling pathways in their kidneys (Fig. 4C), which was consistent with previous KO mice data (Fig. 1). Interestingly, among the top 15 GSEA enrichment terms with lowest *p*-values comparing *Slc3a1* KO males with females, terms related to mitochondrial functions such as oxidation-reduction, fatty acid metabolism, ATP formation, respiratory electron transport, and citric acid cycle were negatively enriched (Fig. 4D). The top 20 biological process and top 20 cellular component terms also indicated significantly decreased mitochondria-related processes in *Slc3a1* KO male kidneys compared with female kidneys (Fig. S3). These data suggest a stronger mitochondrial function in *Slc3a1* KO female kidneys than in male ones.

### The *Slc3a1* KO male kidneys exhibited impairments in mitochondrial functions

With the bulk RNA sequencing results mentioned above, we further investigated the mitochondrial functions in *Slc3a1* KO male and female kidneys. Initially, we examined the changes in mitochondrial biogenetic markers between WT and *Slc3a1* KO mice of the same sex to confirm the involvement of mitochondria in cystinuria. The expression levels of cytochrome c oxidase subunit 4 (*Cox4*) and OXPHOS-related genes (*Ndufb8*, *Sdhb*, *Uqcr2*) were significantly decreased in *Slc3a1* KO mice (Fig. S4A). Additionally, there was a significant decrease in mtDNA/nDNA levels, indicating reduced mitochondrial content in *Slc3a1* KO mice (Fig. S4B). These data suggest that notable mitochondrial defects occur during cystinuria.

**Figure 5:**
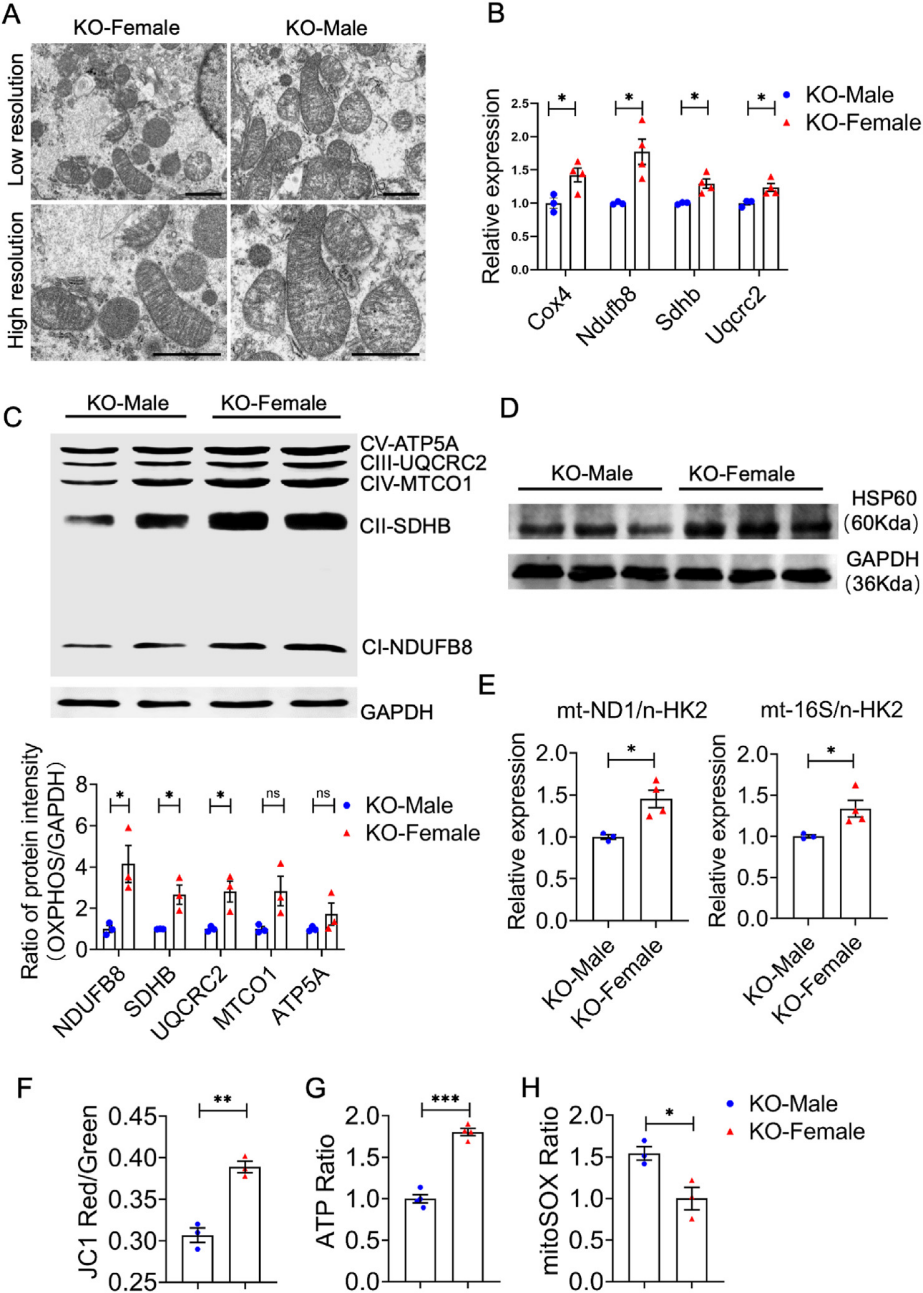
The male kidney of *Slc3a1* KO exhibited impairments in mitochondrial functions. **(A)** Representative transmission electron micrographs of mitochondria in the renal tubule of *Slc3a1* KO male and female mice. Scale bar, 1 μm in the upper panel and 533 nm in the lower panel. **(B)** Relative mRNA levels of *Cox4* and OXPHOS genes (*Ndufb8*, *Sdhb*, and *Uqcrc2*) in the kidneys of *Slc3a1* KO male and female mice. **(C)** Western blots of OXPHOS genes (NDUFB8, SDHB, UQCRC2, MTCO1, and ATR5A) in the kidneys of *Slc3a1* KO male and female mice (upper) and the quantification of their protein levels (lower). **(D)** Western blots of HSP60 in the kidneys of *Slc3a1* KO male and female mice. **(E)** Relative mtDNA levels in the kidneys of *Slc3a1* KO male and female mice using quantitative PCR by amplification of ND1 and 16S genes and normalization against the hexokinase 2 (HK2) gene. **(F–H)** Quantification of fluorescent JC1 signaling (red/green ratio), fluorescent ATP signaling, and fluorescent mitoSOX signaling in primary cells from the kidneys of *Slc3a1* KO male and female mice. All data are represented as mean ± standard error of the mean. *p* values were calculated by *t*-test with the post hoc Tukey test. ∗*p* < 0.05.

Based on these results, we are interested in understanding whether differences in basal conditions without stone formation contribute to observed sex dimorphism regarding mitochondrial defects in *Slc3a1* KO mice. As the size and number of cystine stones varied between sexes, potentially impacting kidney function to varying degrees, we selected 4-week-old *Slc3a1* KO mice that had not developed bladder stones for subsequent experiments. This allowed us to eliminate the influence of varying stone burdens. We first analyzed renal mitochondrial morphology using the transmission electron micrographs in male and female *Slc3a1* KO mice. The results revealed swollen mitochondria with collapsed internal cristae in *Slc3a1* KO males, while females exhibited normal mitochondrial morphology with relatively more organized cristae (Fig. 5A). Consistently, quantitative PCR and Western blot analyses demonstrated lower levels of mitochondrial markers such as COX4 and HSP60, as well as OXPHOS complex-related genes in *Slc3a1* KO males versus females (Fig. 5B–D). Additionally, a lower ratio of mtDNA to nuclear DNA was observed in *Slc3a1* KO males compared with females (Fig. 5E). The role of *Slc3a1* in mitochondrial functions was further investigated by measuring JC-1, ATP, and mitoSOX levels in primary cells from male and female mice lacking *Slc3a1*. The results showed that the ratio of red to green fluorescence of JC-1 was lower in male KO mice, indicating decreased mitochondrial membrane potential (Fig. 5F). Additionally, ATP levels were lower in male KO kidneys compared with females (Fig. 5G), while ROS generation was higher in males as indicated by elevated mitoSOX levels (Fig. 5H). Collectively, these findings suggest that weak mitochondrial functions were present in *Slc3a1* KO males before stone formation and kidney injuries. As mitochondria serve as energy factories within renal tubule cells, this weakened functionality implies improper functioning of renal tubule cells from *Slc3a1* KO males, exacerbating injuries by accumulating more debris and forming cystine crystal-containing stones.

### High SLC3A1 protein level was associated with enhanced mitochondrial functions in the kidney

**Figure 6:**
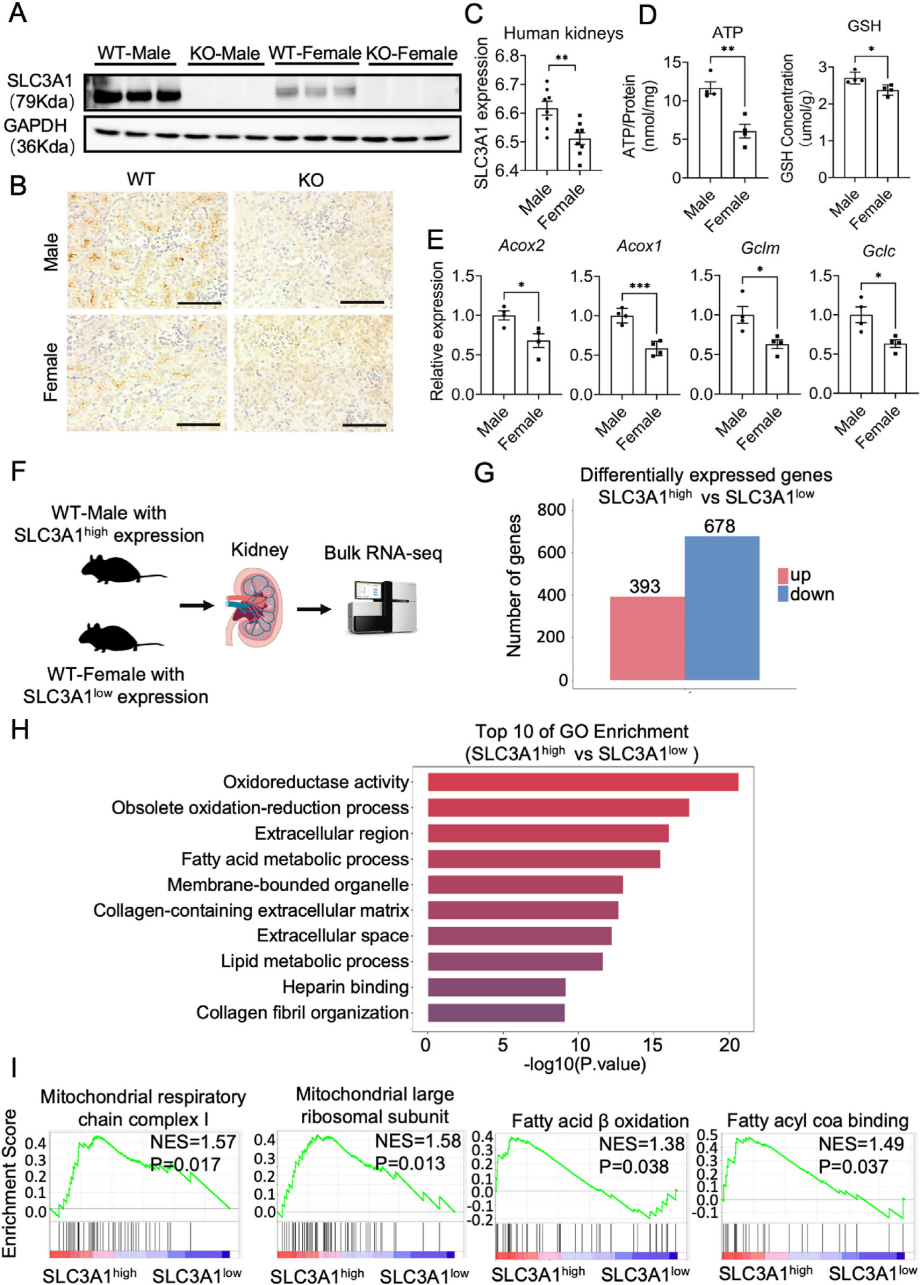
High SLC3A1 protein level was associated with enhanced mitochondrial functions in the kidney. **(A)** Protein levels of SLC3A1 in the kidneys of WT and *Slc3a1* KO mice from both sexes. **(B)** SLC3A1 expression in the kidneys of *Slc3a1* KO and WT mice from both sexes by immunohistochemical staining. Scale bar, 100 μm. **(C)** The relative mRNA expression of *SLC3A1* in the healthy human samples. **(D)** The content of ATP and GSH in the primary tubule cells derived from kidneys of SLC3A1^high^ males and SLC3A1^low^ females. **(E)** Transcript expression of *Acox1*, *Acox2*, *Gclc*, and *Gclm* in the primary tubule cells derived from kidneys of SLC3A1^high^ males and SLC3A1^low^ females. **(F)** Schematic diagram of the bulk RNA sequencing (RNA-req) with kidneys from SLCA31^high^ males and SLCA31^low^ females. **(G)** Differentially expressed genes in bulk RNA-seq data of SLCA31^high^ male kidneys versus SLCA31^low^-female kidneys with 393 up-regulated (red) and 678 down-regulated (blue) genes. **(H)** Top 10 GO enrichment terms of SLCA31^high^-male kidneys over SLCA31^low^-female kidneys from bulk RNA-seq data. **(I)** Gene set enrichment analysis (GSEA) analysis of the mitochondrial and energy metabolism of LCA31^high^ male kidneys over SLCA31^low^ female kidneys from bulk RNA-seq data. All data are represented as mean ± standard error of the mean. *p* values were calculated by *t*-test with the post hoc Tukey test. ∗∗∗*p* < 0.001, ∗∗*p* < 0.01, ∗*p* < 0.05.

To further investigate the relationship between SLC3A1 and sex dimorphism of mitochondrial functions, we compared the protein levels of SLC3A1 in male/female WT and *Slc3a1* KO mice. The results demonstrated complete deletion of SLC3A1 in KO kidneys, regardless of sexes (Fig. 6A). Surprisingly, we observed significantly higher protein levels of SLC3A1 in male WT kidneys compared with female WT kidneys in western blotting and immunohistochemistry assays (Fig. 6A, B), inconsistent with the reported single-cell RNA sequencing and quantitative PCR results (Fig. 2A, B). To confirm SLC3A1's role in human kidneys, we examined its expression in healthy human kidneys from a reported dataset of Nephroseq.^30^ The results showed consistently higher expression of *SLC3A1* in male kidneys versus female kidneys (Fig. 6C). We were intrigued by whether these differences in SLC3A1 expression between males and females could impact mitochondrial functions. Therefore, we cultured primary tubule cells isolated from males with high SLC3A1 expression (SLC3A1^high^) and females with low SLC3A1 expression (SLC3A1^low^), followed by examination of their mitochondrial functions. The results showed that primary tubule cells from male kidneys exhibited elevated GSH and ATP levels, as well as significantly increased transcript expression of fatty acid metabolism-related genes *Acox1* and *Acox2* and GSH metabolism-related genes *Gclc* and *Gclm* (Fig. 6D, E). These observations suggested that male kidneys with high SLC3A1 expression required more energy than female kidneys for maintaining physiological activity at baseline. To gain further insights into the mitochondrial functions within both types of kidneys (SLC3A1^high^ male versus SLC3A1^low^ female), unbiased bulk RNA sequencing was performed on kidney samples (Fig. 6F). Analysis revealed 1071 differentially expressed genes, including 393 up-regulated genes and 678 down-regulated genes when comparing SLCA31^high^ male versus SLCA31^low^ female kidneys (Fig. 6G). Notably, the differentially expressed genes identified in this study were predominantly associated with oxidation and fatty acid metabolism, as revealed by the GO enrichment analysis (Fig. 6G). Specifically, SLC3A1^high^ male kidneys exhibited a positive correlation with mitochondrial respiratory chain complex 1, mitochondrial large ribosomal subunit, fatty acid beta oxidation, and fatty acyl coa binding (Fig. 6H). Overall, these findings suggest that SLC3A1^high^ male kidneys require increased energy and more active mitochondria to maintain their physiological activity under normal conditions. Conversely, the absence of SLC3A1 renders male kidneys more vulnerable to injuries.

### Elevated SLC3A1 protein level enhanced mitochondrial functions in proximal tubule cells

**Figure 7:**
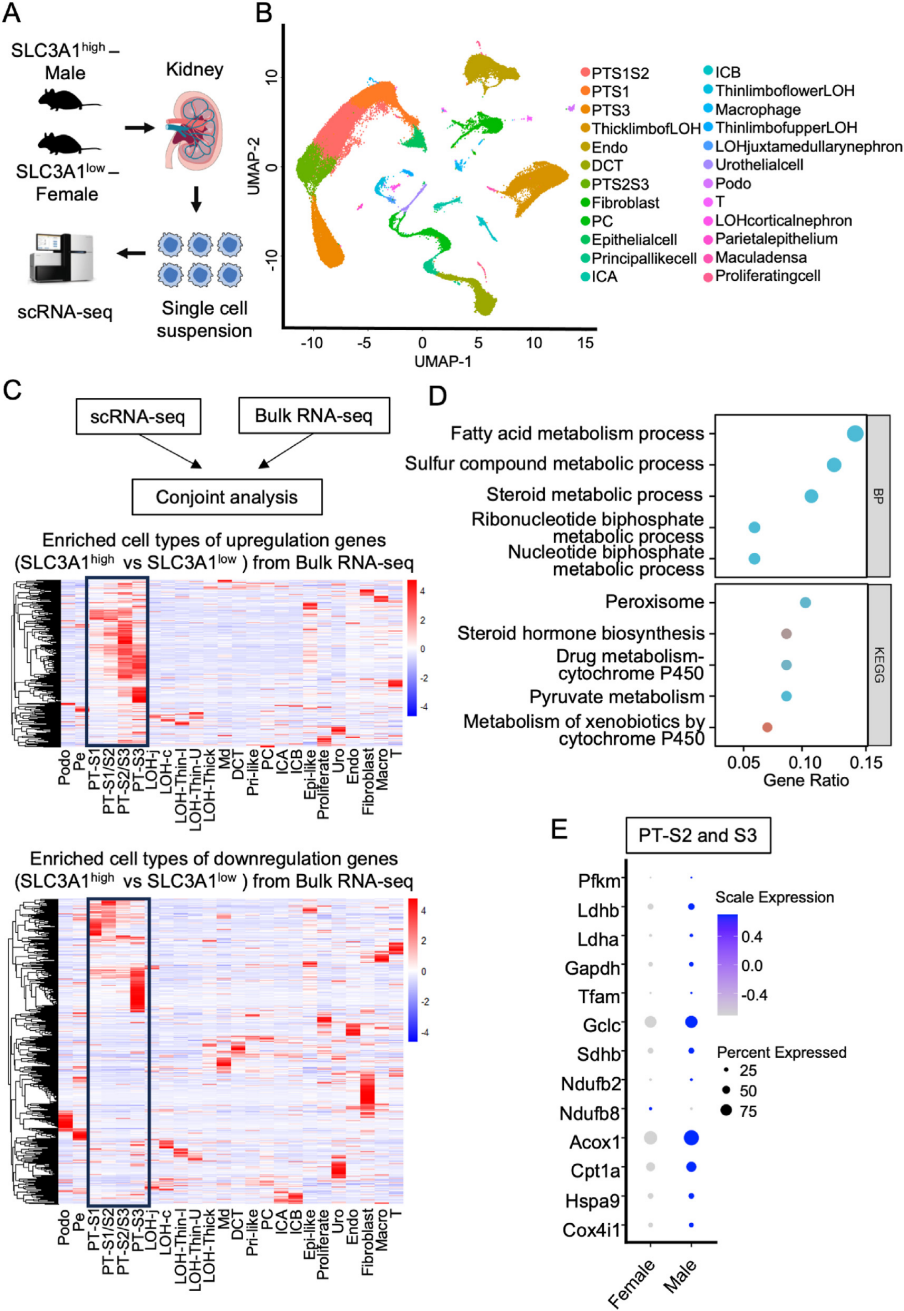
Elevated SLC3A1 protein level was associated with enhanced mitochondrial functions in proximal tubule cells. **(A)** Schematic diagram of the single-cell RNA sequencing (scRNA-seq) with kidneys from SLCA31^high^ males and SLCA31^low^ females. **(B)** The UMAP of 24 distinct cell types identified by unsupervised clustering. Podo, podocyte; Pe, parietal epithelium; PT-S1, S1 segment of proximal tubule; PT-S1/S2, S1 and S2 segments of proximal tubule; PT-S2/S3, S2 and S3 segments of proximal tubule; PT-S3, S3 segment of proximal tubule; LOH-j, LOH of juxtamedullary nephron; LOH-c, LOH of cortical nephron; LOH-Thin-U, thin limb of upper LOH; LOH-Thin-L, thin limb of lower LOH; LOH-Thick, thick limb of LOH; Md, macula densa; DCT, distal convoluted tubule; Pri-like, principal like cell; PC, principal cell; ICA, intercalated type A cell; ICB, intercalated type B cell; Epi, epithelial cell; Pro, proliferating cell; Uro, urothelial cell; Endo, endothelial cell; Fibro, fibroblast; Macro, macrophage; T, T cell. **(C)** The workflow of the integration of scRNA-seq and bulk RNA-seq with kidneys from SLCA31^high^ males and SLCA31^low^ females (upper) and cell-type-specific expression of top differentially expressed genes (DEGs) identified in (B) in the scRNA-seq dataset (middle and lower). The mean expression values of the genes were calculated in each cluster. The color scheme is based on Z score distribution. **(D)** GO enrichment terms of PT-specific DEGs. **(E)** Bubble plots of mitochondrial functions and energy production related genes in S2 and S3 segments of PT from SLCA31^high^ males and SLCA31^low^ females.

As the kidney has been demonstrated as a high heterogeneity organ,^29^ we then performed single-cell RNA sequencing on SLC3A1^high^ and SLC3A1^low^ kidneys to further investigate the impact of SLC3A1 on kidney metabolism at single-cell resolution (Fig. 7A). Six samples were analyzed, including three SLC3A1^high^ male kidneys and three SLC3A1^low^ female kidneys. Cell clustering analysis revealed 24 major types of cells, including kidney epithelial cells, immune cells, and endothelial cells based on the expression patterns of marker genes (Fig. 7B; Fig. S5A). Integrating bulk RNA sequencing data (Fig. 6) with single-cell RNA sequencing data, we observed that significantly changed genes between males and females, which were enriched in the signaling pathway related to mitochondrial functions, showed enrichment for proximal tubule cells (Fig. 7C, D). From the integrative analysis, we found that S2 and S3 segments of proximal tubule cells are the cell types with the strongest transcriptomic changes (Fig. 7C), implying a significant contribution of S2 and S3 segments of proximal tubule cells in orchestrating transcriptomic alterations within SLC3A1^high^ and SLC3A1^low^ kidneys. Specifically, mitochondria function and energy production related genes are more enriched in the S2 and S3 segments than the S1 segment of proximal tubule cells in SLC3A1^high^ male kidneys (Fig. 7E; Fig. S4B). In summary, the differential mitochondrial functions between SLC3A1^high^ male kidney and SLC3A1^low^ female kidney primarily arise from proximal tubule cells.

### SLC3A1 modulated mitochondrial NAD^+^ uptake

**Figure 8:**
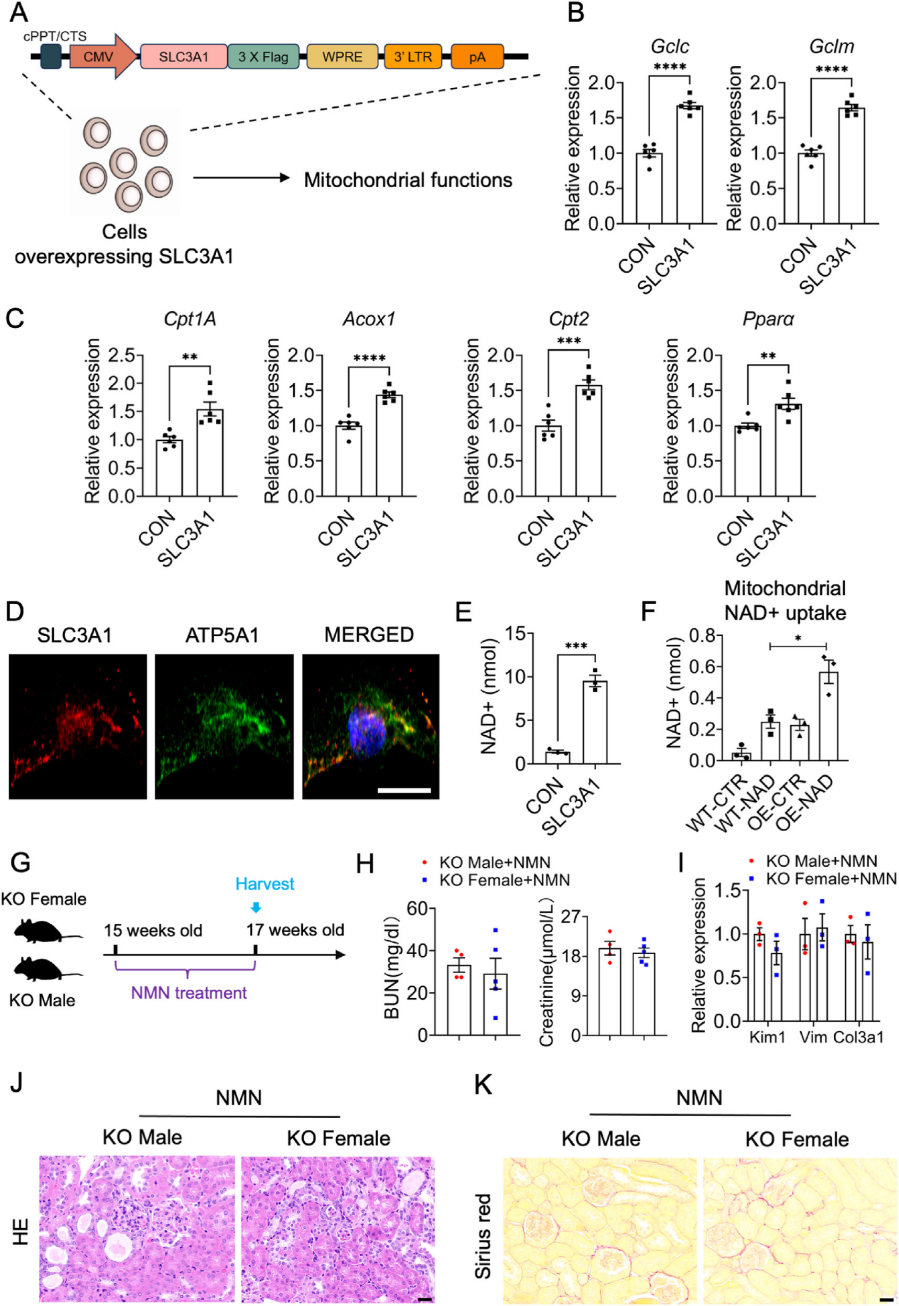
SLC3A1 mediated mitochondrial functions by NAD ^+^ uptake. **(A)** Schematic diagram of SLC3A1 overexpression experiments. **(B)** Transcript expression of GSH metabolism related genes *Gclc* and *Gclm* in the transfected or non-transfected cells. **(C)** Transcript expression of fatty acid metabolism related genes *Cpt1a*, *Acox1*, *Cpt2*, and *Acox2* in the transfected or non-transfected cells. **(D)** Representative images of double staining of SLC3A1 and the mitochondrial marker ATP5A1 in HK2 cells. Scale bar, 10 μm. **(E)** NAD^+^ content of control and SLC3A1 overexpressed cells. **(F)** NAD^+^ content of mitochondria isolated from HEK 293T control cells and cells stably overexpressing (OE) SLC3A1 before and after a 20-min incubation with 1 mM NAD^+^. **(G)** Scheme of the experimental approach. The mice were intraperitoneally injected with NMN (500 mg/kg) and then euthanized after 14 days. **(H)** Serum blood urea nitrogen (BUN) of *Slc3a1* KO mice from both sexes with NMN supplement. **(I)** Relative mRNA levels of fibrosis and inflammatory genes (*Kim1*, *Vimentin*, and *Col3a1*) in the kidneys of *Slc3a1* KO mice from both sexes with NMN supplement. **(J, K)** Representative images of hematoxylin/eosin- and Sirius red-stained kidney sections of *Slc3a1* KO mice from both sexes with NMN supplement. Scale bar, 100 μm. All data are represented as mean ± standard deviation of the mean. *p* values were calculated by *t*-test with the post hoc Tukey test. ∗∗∗∗*p* < 0.0001, ∗∗∗*p* < 0.001, ∗∗*p* < 0.01, ∗*p* < 0.05.

To demonstrate that *Slc3a1* indeed increases the functions of mitochondria, we transfected *Slc3a1*-expressing construct to the HEK293T cell (Fig. 8A). The results showed that *Slc3a1* overexpressed cells exhibited elevated transcript expression of fatty acid metabolism-related genes *Cpt1a*, *Acox1*, *Cpt2*, and *Acox2*, and GSH metabolism-related genes *Gclc* and *Gclm* (Fig. 8B, C). These observations suggested that SLC3A1 enhanced mitochondrial functions at baseline.

To further investigate how SLC3A1 regulates mitochondrial functions, we initially examined its cellular location. Our observations in HK2 cells revealed that SLC3A1 colocalized with the mitochondrial marker ATP5A1 (Fig. 8D), consistent with the reported subcellular location of SLC3A1 on The Human Protein Atlas website. Considering SLC3A1's role as a transporter, we hypothesized that it might be responsible for transporting necessary factors for mitochondrial functions. To test this hypothesis, we overexpressed SLC3A1 and observed a significant increase in cellular NAD^+^ levels (Fig. 8E), suggesting that SLC3A1 may facilitate mitochondrial NAD^+^ uptake. To verify this, we conducted a mitochondrial NAD^+^ uptake experiment by isolating mitochondria from control and SLC3A1 overexpressed cells and incubating them with exogenous NAD^+^. Cells were treated with FK866 to inhibit NAD^+^ synthesis. Exogenous NAD^+^ significantly increased matrix NAD^+^ content in mitochondria from SLC3A1 overexpressed cells compared with control cells (Fig. 8F), suggesting that SLC3A1 mediates the uptake of NAD^+^ into mammalian mitochondria.

To test the mechanism *in vivo*, we treated female and male SLC3A1 KO mice with a NAD^+^ precursor, NMN, for two weeks (Fig. 8G), to determine if it could rescue the sex differences of cystinuria. The results showed that after NMN supplementation, serum blood urea nitrogen and creatinine levels were comparable between female and male KO mice (Fig. 8H). As expected, there were no significant differences in fibrotic and injury markers such as *Kim1*, *Vimentin*, and *Col3a1* between female and male KO mice (Fig. 8I). In addition, hematoxylin and eosin staining and Sirius red staining did not show more tubular injury or greater degree of fibrosis in *Slc3a1* KO male mice with NMN supplementation compared with females (Fig. 8J, K). Overall, these findings suggest that SLC3A1 mediates NAD^+^ uptake into mitochondria and the NAD^+^ precursor NMN mitigates the sex difference in *Slc3a1* KO mice.

## Discussion

The lack of understanding of the molecular mechanisms underlying sex dimorphism in cystinuria has been a significant obstacle to comprehending the differences between sexes in this condition, impeding the development of new drugs and their proper utilization. In this study, we employed unbiased bulk and single-cell RNA sequencing to analyze a well-established mouse model known as the *Slc3a1* KO model, which provides an invaluable resource for the scientific community. Consistent with previous findings,^31^ our results demonstrate that male *Slc3a1* KO mice exhibit more severe phenotypes compared with females, including increased stone formation and size, elevated blood urea nitrogen, and greater tubule dilations. These effects were not alleviated by double KO of the sex-dependent-expressed cystine transporter *Slc7a13* or orchidectomy in single KO *Slc3a1* mice. By integrating unbiased bulk RNA and single-cell sequencing data, our investigation indicates that SLC3A1 protein levels are associated with mitochondrial functions in renal proximal tubule cells. Increased expression of SLC3A1 leads to enhanced mitochondrial functions by modulation of mitochondrial NAD^+^ uptake mainly in male proximal tubules, which has not been reported previously.

Concerns regarding sex differences are increasingly relevant in scientific and societal domains, yet there are still gaps in our understanding of the mechanisms underlying sex disparities in renal pathophysiology, disease progression, and management.^32^ Growing evidence has demonstrated that while male hormones have a detrimental effect by increasing oxidative stress, activating the RAS system, and exacerbating fibrosis within the damaged kidney, female hormones exert a renoprotective effect.^33^ Considering the significant sex bias of cystinuria and the implicated functions of male hormones, we performed an orchidectomy procedure on *Slc3a1* KO male mice. In contrast to the role of androgens in accelerating chronic kidney disease,^34^ we did not observe a significant difference between normal *Slc3a1* KO males and orchiectomized *Slc3a1* KO males. Our findings align with clinical observations for cystinuria where male specificity is evident from childhood, suggesting that sex hormones are unlikely to play a substantial role in the development of cystinuria.^35^

Mechanistically, our studies suggest that mitochondria are a central target for the sexual dimorphism of cystinuria. Sexual dimorphism in mitochondrial impairment exists in the occurrence and development of numerous diseases, such as sepsis-induced acute kidney injury, cardiovascular disorders, metabolic disorders, neurological disorders, and aging.^18^^,^^36^ Proximal renal tubules play a critical role in reabsorbing essential substrates filtered from the blood and excreting waste and toxins into the urine, which energetically requires high mitochondrial density. We noted that SLC3A1 protein is predominantly located in proximal tubules and high levels of SLC3A1 protein in male kidneys were associated with enhanced mitochondrial functions in WT male kidneys, indicating that WT male kidneys require more energy to maintain physiological activity under basal conditions. Cystine, which is involved in GSH synthesis, serves as an energy source within cells, therefore its deficiency is associated with impaired mitochondrial functions.^9^ When cystine transporters such as SLC3A1 are absent in the kidneys, the transportation of extracellular cystine into the cells is hindered, resulting in renal cell dysfunction caused by mitochondrial impairment. Similar to the condition that WT proximal tubules require high mitochondrial density, kidney tubule cells are vulnerable to injuries caused by hypoxia or toxin,^37^^,^^38^ thus WT male kidneys need more robust mitochondrial functions which make them susceptible to disease when cystine transporter SLC3A1 is missing. Consequently, *Slc3a1* KO male mice exhibit lower mitochondrial functions and more severe tubule damage. The field of crystal-renal cell interaction has witnessed extensive research, which highlights the crucial role played by cell fragments, such as impaired brush border membrane^39^^,^^40^ and infectious substances,^41^ in facilitating crystal nucleation and adhesion. Our studies have revealed that kidney injury is more severe in *Slc3a1* KO males compared with females, manifesting as increased cell apoptosis, inflammatory cell infiltration, and collagen fiber deposition. These sex differences may serve as heterogeneous foci for initiating nucleation and promoting the adhesion and retention of crystals.

NAD^+^ serves as a vital coenzyme in the electron transport chain, playing a crucial role in mitochondrial functions. Numerous research groups have reported that NAD^+^ deficiency is a characteristic of kidney diseases, with studies demonstrating the protective role of NAD ^+^ replacement.^42^ A recent study reveals consistent disruption of NAD ^+^ synthesis in experimental mouse models and patients with kidney disorders.^43^ The researchers further demonstrated that supplementation with NAD ^+^ precursors, such as nicotinamide riboside or nicotinamide, effectively prevented tubular injury in mice by restoring mitochondrial functions. In our study, we demonstrate that *Slc3a1* mediates mitochondrial functions by modulating NAD ^+^ uptake. When NAD^+^ is mutant in cystinuria, mitochondria turn out to be dysfunctional because of the limited cellular NAD^+^ content. To rescue this, we supplemented mice with NAD^+^ precursor NMN, which bypasses cytoplasmic NAD^+^ uptake but maintains mitochondrial NAD^+^ levels. Our study showed that NMN supplementation could mitigate the sex differences observed in cystinuria. The role of NAD ^+^ supplementation in humans has been the subject of several ongoing studies.^44^ A clinical trial investigated the impact of oral nicotinamide riboside treatment on patients with chronic kidney disease, revealing that a 6-week administration of nicotinamide riboside effectively enhanced and modified plasma metabolic and lipidomic profiles.^45^ Our study suggests that supplementing with NAD ^+^ precursors may be a potential intervention strategy for cystinuria.

In conclusion, our study provides a comprehensive and unbiased dataset of bulk and single-cell RNA sequencing, highlighting the role of mitochondrial functions in the sex dimorphism of cystinuria. Our findings suggest a potential link between the restoration of mitochondria in renal tubules of male cystinuria patients and the improvement of mitochondrial functions, leading to reduced cell death and attenuation of fibro-inflammation in renal tubules. Future research should focus on investigating more interventions for the sex-dependent phenotype of cystinuria, specifically targeting mitochondrial function restoration.

## CRediT authorship contribution statement

**Jingyi Su:** Conceptualization, Investigation, Methodology, Project administration, Validation, Writing – original draft. **Yongdong Pan:** Data curation, Methodology. **Fengbo Zhong:** Methodology. **Yi Zhong:** Methodology. **Jiaxin Huang:** Data curation. **Shengnan Liu:** Methodology. **Kaiyuan Wang:** Data curation. **Kai Lin:** Data curation. **Xiangchen Gu:** Methodology. **Dali Li:** Resources. **Qihui Wu:** Methodology. **Hongquan Geng:** Resources. **Yuting Guan:** Supervision. **Guofeng Xu:** Project administration, Resources, Supervision, Writing – review & editing.

## Ethics declaration

All experiments were conducted in accordance with the guidelines of the Animal Welfare Act for the care and use of laboratory animals, and all the protocols were approved by the Xinhua Hospital Animal Care and Use Committee.

## Funding

This work is supported by grants from the 10.13039/501100003399Science and Technology Commission of Shanghai Municipality of China (No. 23Y21900102 to G.X.; 23ZR1467900 to Q.W.), 10.13039/501100013105Shanghai Rising-Star Program (No. 22QA1405900 to Y.G.), the 10.13039/501100012166National Key R&D Program of China (No. 2022YFC2505400, 2022YFC3400203 to Y.G.), the 10.13039/501100001809National Natural Science Foundation of China (No. 82100773 to Y.G.; 82101486, 82371426 to Q.W.), the 10.13039/501100005230Natural Science Foundation of Chongqing, China (No. CSTB2022NSCQ-MSX1621 to Y.G.), the Ningxia Hui Autonomous Region Key Research and Development Project (China) (No. 2022BFH02012 to Q.W.).

## Conflict of interests

The authors declared no competing interests.
