# Supplementary material for: Mitochondrial SLC3A1 regulates sexual dimorphism in cystinuria
Source: Genes Dis. 2024 Nov 29;12(3):101472. doi: 10.1016/j.gendis.2024.101472 (PMC11919626; doi:10.1016/j.gendis.2024.101472)
Supplement: Multimedia component 1 [file mmc1.pdf]

Supplementary Materials for

Mitochondrial SLC3A1 regulates sexual dimorphism in cystinuria

Jingyi Su[a,#], Yongdong Pan[a,#], Fengbo Zhong[b,#], Yi Zhong[b], Jiaxin Huang[a], Shengnan Liu[a], Kaiyuan Wang[b], Kai Lin[b], Xiangchen Gu[c], Dali Li[b], Qihui Wu[d], Hongquan Geng[e, *], Yuting Guan[a,b,f,**], Guofeng Xu[a,***]

Correspondence authors:

*Hongquan Geng. Department of Urology, Children's Hospital of Fudan University, Shanghai, 201102, China. E-mail: genghongquan@fudan.edu.cn

**Yuting Guan. Shanghai Frontiers Science Center of Genome Editing and Cell Therapy, Shanghai Key Laboratory of Regulatory Biology, Institute of Biomedical Sciences and School of Life Sciences, East China Normal University, Shanghai, 200241, China. E-mail: ytguan@bio.ecnu.edu.cn

***Guofeng Xu. Department of Pediatric Urology, Xinhua Hospital Affiliated to Shanghai Jiao Tong University School of Medicine, Shanghai, 200092, China. E-mail: xuguofeng@xinhuamed.com.cn

This PDF file includes:

Figs. S1 to S5

Tables S1 to S3

Figure S1

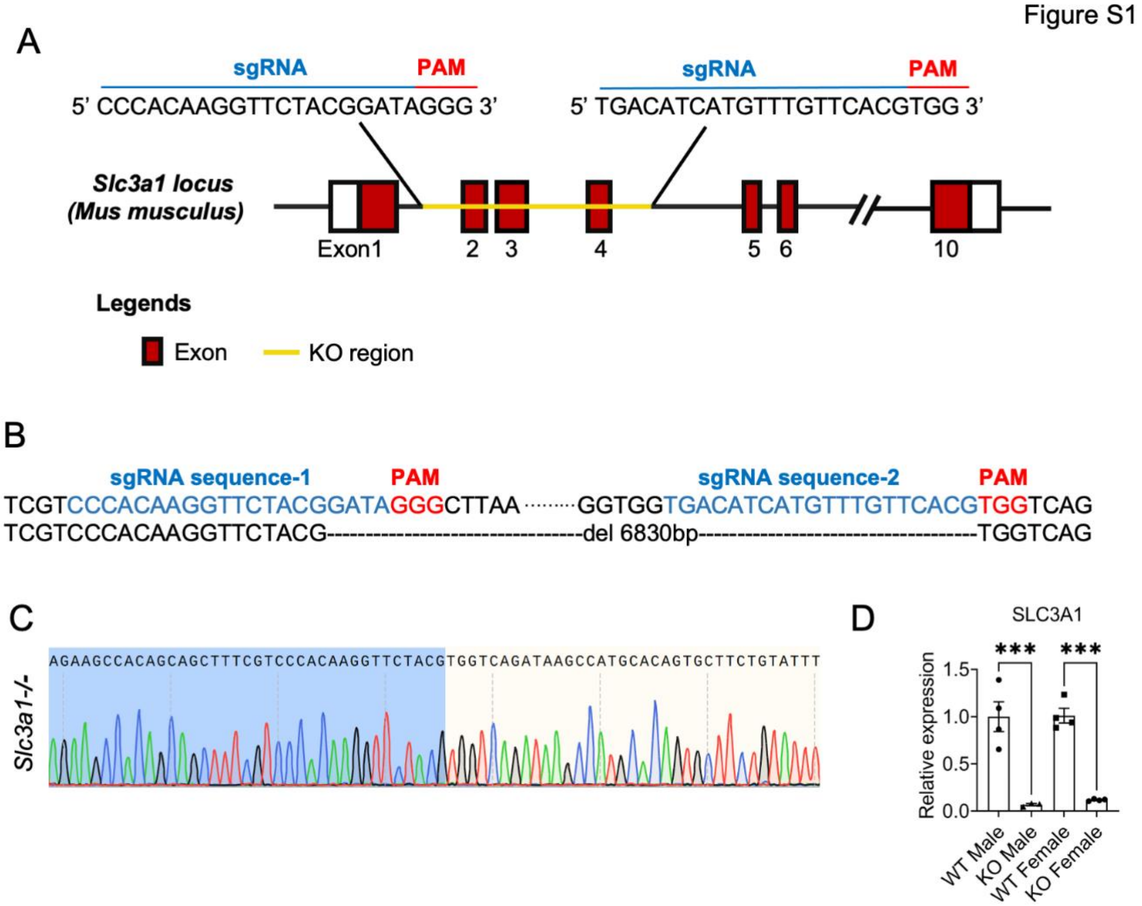

Fig. S1. Generation and identification of *Slc3a1* KO mouse.

A. Targeting strategy of *Slc3a1* KO mouse model using CRISPR-Cas9.

B. The targeted sgRNA sequence designed for the CRISPR-Cas9 system. The dashed lines stand for deletion nucleotides.

C. Sequencing result of homozygous *Slc3a1* KO mouse.

D. Relative mRNA levels of *Slc3a1* in the kidneys of *Slc3a1* KO and WT mouse from both genders. ***$p < 0.001$.

Figure S2

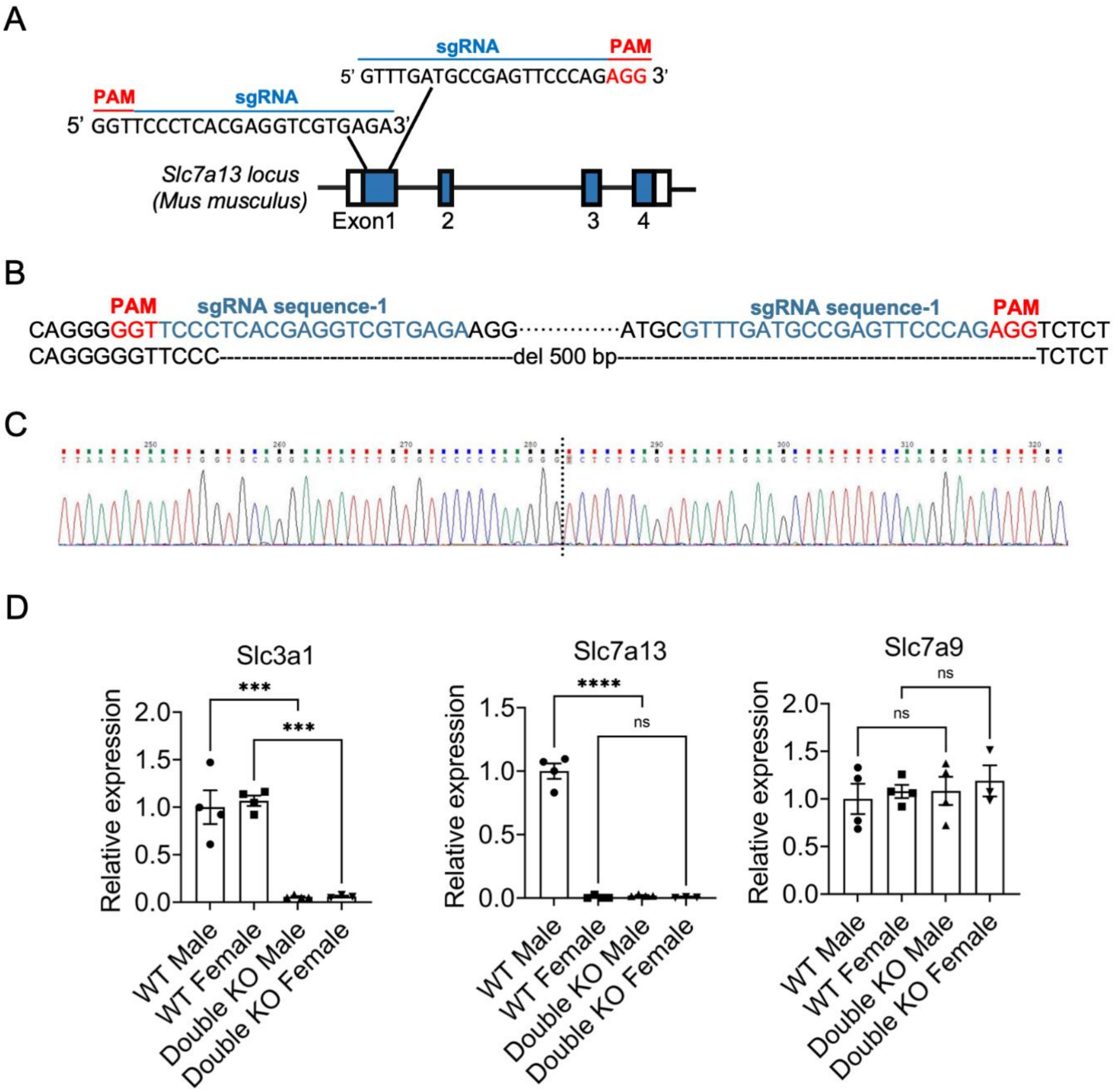

Fig. S2. Generation and identification of *Slc7a13* KO mouse.

A. Targeting strategy of *Slc7a13* KO mouse model using CRISPR-Cas9/sgRNA system.

B. The targeted sgRNA sequence designed for the CRISPR-Cas9/sgRNA system. The dashed line stands for deletion nucleotides.

C. Sanger sequencing result of homozygous *Slc7a13* KO mouse.

D. Relative mRNA levels of *Slc3a1*, *Slc7a13* and *Slc7a9* in the kidneys of WT and DKO mouse from both genders. ns, no significant difference; ****, p<0.0001; ***, p < 0.001.

Figure S3

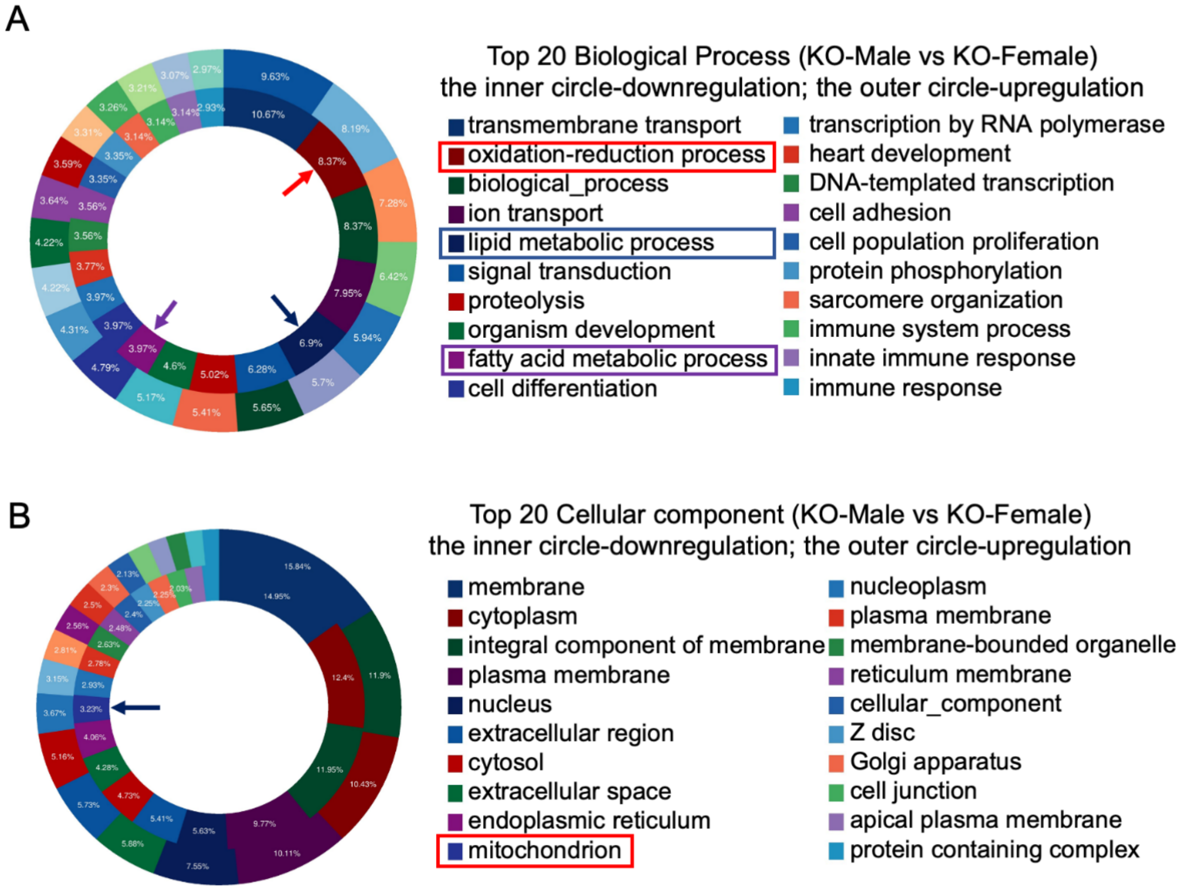

Fig. S3. The Gene Ontology enrichment analysis between *Slc3a1* KO males and females.

A. Top 20 Biological Process analysis of SLCA31^{high}-male kidneys over SLCA31^{low}-female kidneys from bulk RNA-seq data.

B. Top 20 Cellular Component terms of SLCA31^{high}-male kidneys over SLCA31^{low}-female kidneys from bulk RNA-seq data.

Figure S4

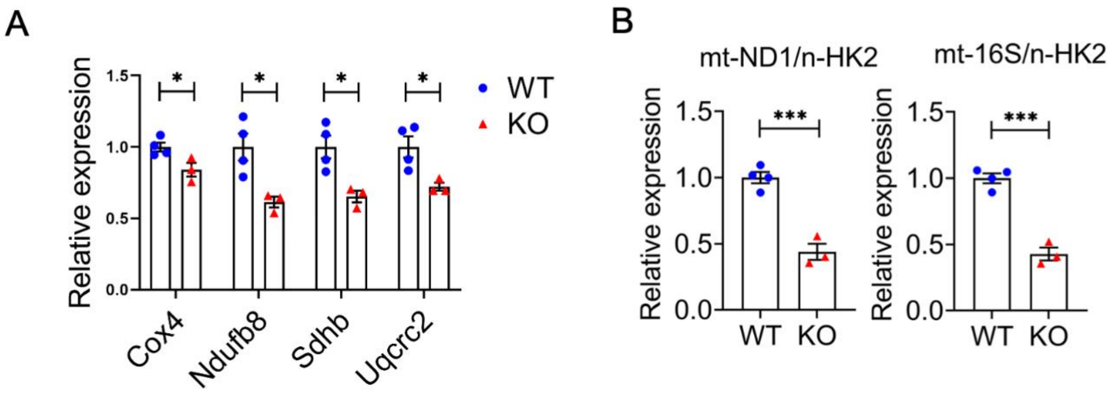

Fig. S4. Mitochondrial assessment in the kidneys of WT and *Slc3a1* KO mice.

A. Relative mRNA levels of *Cox4* and OXPHOS-related genes (*Ndufb8*, *Sdhb*, and *Uqcrc2*) in the kidneys of *Slc3a1* KO and WT mice.

B. Relative mtDNA levels in WT and *Slc3a1* KO mice kidney using qPCR by amplification of ND1 and 16S genes and normalization against the hexokinase 2 (HK2) gene; *, $p<0.05$; ***, $p < 0.001$.

Figure S5

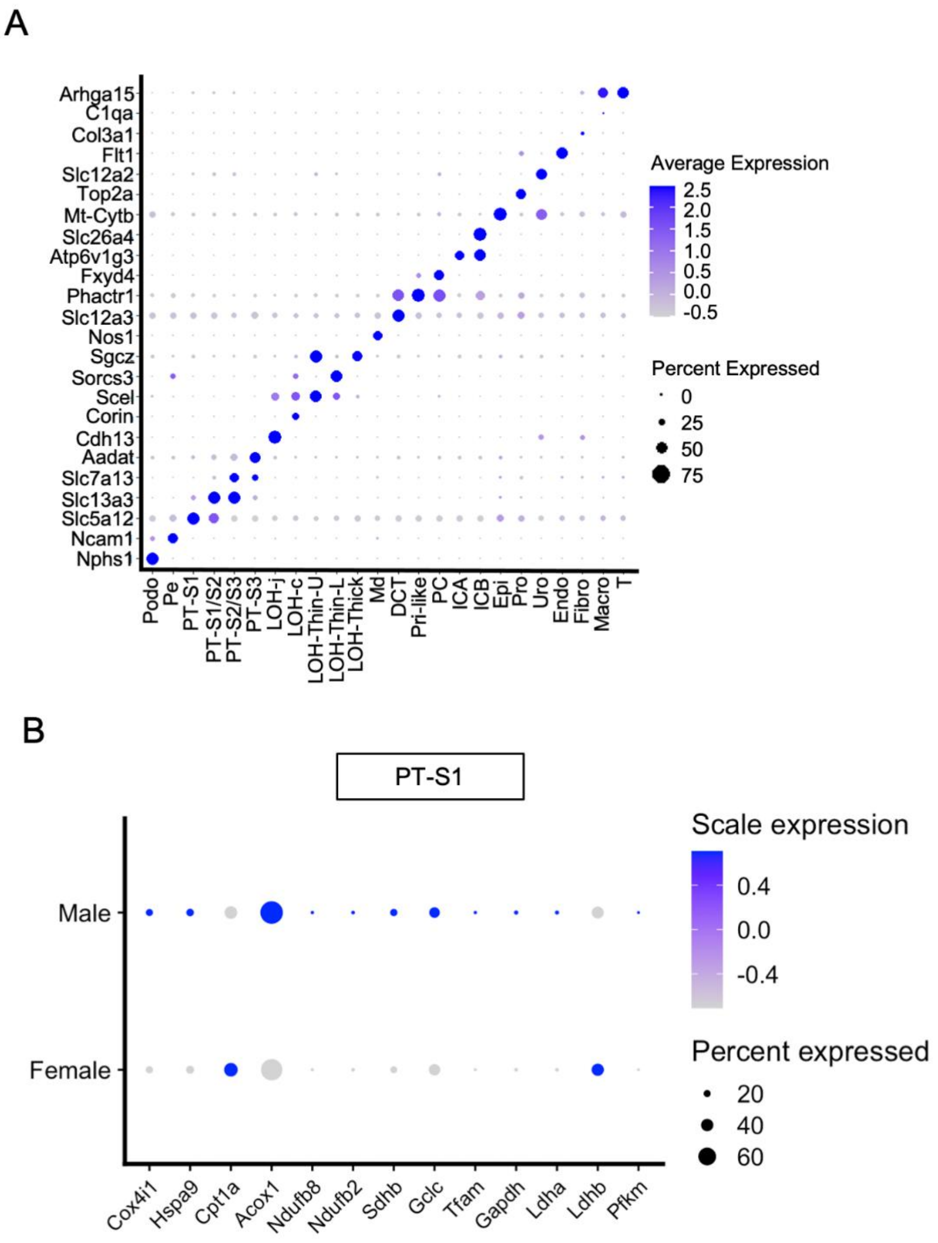

Fig. S5. Single cell RNA sequencing of SLC3A1^{high} male kidney and SLC3A1^{low} female kidney.

A. Bubble plots of cell cluster marker genes identified in SLC3A1^{high} male kidney and SLC3A1^{low} female kidney samples.

B. Bubble plots of mitochondrial functions and energy production related genes in S2 and S3 of PT from SLCA31^{high}-males and SLCA31^{low}-females.

Table S1. sgRNA and identification primer sequence

**For deletion of *Slc3a1* gene in mice**	
Slc3a1-sgRNA-1	AGAGTGCTGGAGCACTCCCTTGG
Slc3a1-sgRNA-2	GTTTGATGCCGAGTTCCCAGAGG
**For deletion of *Slc7a13* gene in mice**	
Slc7a13-sgRNA-1	GGTTCCCTCACGAGGTCGTGAGA
Slc7a13-sgRNA-2	GTTTGATGCCGAGTTCCCAGAGG
**For genotyping of *Slc3a1* mutant mice**	
Slc3a1-F	AGGAGATTCTGGAGGAAAATGTCA
Slc3a1-R	CCCTTCTGTGGAAGAGCTGGTT
**For genotyping of *Slc7a13* mutant mice**	
Slc7a13-F	AGGGTCAAACTAAGCCCTTACAG
Slc7a13-R	TGGAATGGATTGAACGGGATGAA

Table S2. qPCR primer sequences

**Mouse Primers**	
Slc3a1-F	ACATGCTCCTGTTCACACTC
Slc3a1-R	ACTATTGTCCCACTGCATCG
Slc7a13-F	GATACTTTGCGTTTTCTGGCG
Slc7a13-R	ACTGTCAGGTAGGAAATATTAGCC
Slc7a9-F	TTGTCATTGGGATTCCTCTGG
Slc7a9-R	AAGATGCTGGATAGAGAACGC
Col3a1-F	ACAGCTGGTGAACCTGGAAG
Col3a1-R	ACCAGGAGATCCATCTCGAC
Fibronectin-F	ACAAGGTTCGGGAAGAGGTT
Fibronectin-R	CCGTGTAAGGGTCAAAGCAT
Vimentin-F	GGAGGCCACGAACTTCACTCT
Vimentin-R	GGGATGCAACACCTATTGTCAGT
Il-1β-F	GCAACTGTTCCTGAACTCAACT
Il-1β-R	ATCTTTTGGGGTCCGTCAACT
Tnf-α-F	CTTCTGTCTACTGAACTTCGGG
Tnf-α-R	CAGGCTTGTCACTCGAATTTTG
Cox4-F	TGAATGGAAGACAGTTGTGGG
Cox4-R	GATCGAAAGTATGAGGGATGGG

Ndufb8-F	ACATCTCTTCGGCTTTGTGG
Ndufb8-R	CAGGCTCTTTGGTAGGATCAC
Sdhb-F	ACCCCTTCTCTGTCTACCG
Sdhb-R	AATGCTCGCTTCTCCTTGTAG
Uqcrc2-F	TTCCAGTGCAGATGTCCAAG
Uqcrc2-R	CTGTTGAAGGACGGTAGAAGG
Acox2-F	AACACAGCATACACAGACCC
Acox2-R	GTCTAGTTTCTCCACAGCTTCC
Acox1-F	CATATGACCCCAAGACCCAAG
Acox1-R	CATGTAACCCGTAGCACTCC
Gclm-F	AATCAGCCCCGATTTAGTCAG
Gclm-R	CGATCCTACAATGAACAGTTTTGC
Gclc-F	ACCATCACTTCATTCCCCAG
Gclc-R	TTCTTGTTAGAGTACCGAAGCG
GAPDH-F	ATGCCAAAGTTGTCATGGAT
GAPDH-R	ATGTTTGTGATGGGTGTGAA
mt-16S-F	CCGCAAGGGAAAGATGAAAGAC
mt-16S-R	TCGTTTGGTTTCGGGGTTTC
mt-ND1-F	CTAGCAGAAACAAACCGGGC
mt-ND1-R	CCGGCTGCGTATTCTACGTT
n-HK2-F	GCCAGCCTCTCCTGATTTTAGTGT
n-HK2-R	GGGAACACAAAAGACCTCTTCTGG

Table S3. Antibodies Lists.

Product Name	Company	Catalog Number	Dilution
SLC3A1	Proteintech	16343-1-AP	1:1000
KIM-1	R&D	AF1817-SP	1:1000
Total OXPHOS Rodent WB antibody Cocktail	abcam	ab110413	1:1000
TOM20	Proteintech	11802-1-AP	1:2000
